# Impact of the COVID-19 pandemic on infectious disease hospitalizations of neonates at a tertiary academic hospital: a cross-sectional study

**DOI:** 10.1186/s12879-022-07211-x

**Published:** 2022-03-02

**Authors:** Jiarong Pan, Canyang Zhan, Tianming Yuan, Yi Sun, Weiyan Wang, Lihua Chen

**Affiliations:** Department of Neonatology, The Children’s Hospital, Zhejiang University School of Medicine, National Clinical Research Center for Child Health, 3333 Binsheng Road, Hangzhou, 310052 Zhejiang People’s Republic of China

**Keywords:** Infection, Neonatology, Inpatient, COVID-19, Epidemiology

## Abstract

**Background:**

To investigate the impact of the coronavirus disease 2019 (COVID-19) pandemic on hospitalizations for neonatal infectious diseases.

**Methods:**

We analyzed data for neonatal inpatients admitted at a tertiary academic hospital with a principal diagnosis of an infectious disease during January 2015 to December 2020. We compared hospitalizations in 2020 (COVID-19 cohort), corresponding with the impact of COVID-19 pandemic and associated containment measures, and the comparable 2015 to 2019 (pre-COVID-19 cohort).

**Results:**

14,468 cases admitted for neonatal infectious diseases were included in our study, with 1201 cases in the COVID-19 cohort and 13,267 cases in the pre-COVID-19 cohort. The leading causes of hospitalizations for neonatal infectious diseases remain being respiratory tract infections (median ratio = 0.461, 95% CI 0.335–0.551), sepsis (median ratio = 0.292, 95% CI 0.263–0.361), gastric intestinal infections (median ratio = 0.095, 95% CI 0.078–0.118) and dermatologic infections (median ratio = 0.058, 95% CI 0.047–0.083). The seasonality of neonatal infectious disease hospitalizations could be obviously observed, with the total number and the overall rate of hospitalizations for neonatal infectious diseases in the first and fourth quarters greater than that of hospitalizations for neonatal infectious diseases in the second and third quarters in each year (1362.67 ± 360.54 vs 1048.67 ± 279.23, P = 0.001; 8176/20020 vs 6292/19369, P < 0.001, respectively). Both the numbers and the proportions of hospitalizations for neonatal infectious diseases in different quarters of the COVID-19 cohort significantly decreased as compared with those forecasted with the data from the pre-COVID-19 cohort: the numbers per quarter (300.25 ± 57.33 vs 546.64 ± 100.43, P-value = 0.006), the first quarter (0.34 vs 0.40, P = 0.002), the second quarter (0.24 vs 0.30, P = 0.001), the third quarter (0.24 vs 0.28, P = 0.024), and the fourth quarter (0.29 vs 0.35, P = 0.003).

**Conclusions:**

Despite the outbreak of the COVID-19 pandemic, the leading causes of hospitalizations for neonatal infectious diseases remain unchanged. The seasonality of neonatal infectious disease hospitalizations could be obviously observed. The numbers as well as the overall rates of hospitalizations for neonatal infectious diseases in the COVID-19 cohort dramatically declined with the impact of the COVID-19 pandemic and its mitigation measures.

**Supplementary Information:**

The online version contains supplementary material available at 10.1186/s12879-022-07211-x.

## Background

Infectious diseases have traditionally been a major cause of morbidity and mortality in neonates [1–3], constituting one of the highest proportions of hospitalization during neonatal period [4]. Acquiring a better understanding of neonatal infectious diseases is crucial for decision making on public health priorities and appropriate management providing for newborn infants. Thus, it is very important to study the status and trends of infectious diseases in neonates.

The coronavirus disease 2019 (COVID-19) global pandemic is caused by a novel coronavirus named severe acute respiratory syndrome coronavirus 2 (SARS-CoV-2), which was first identified in December 2019 [5]. In 2020, as the COVID-19 rapidly developed into a global health emergency, national and local governments around the world introduced mandated lockdowns, quarantines, isolation, mask wearing, social distancing, and other measures to curb the pandemic in the interest of public health [6–8]. Although China has effectively reversed the pandemic situation and curbed the spread of COVID-19 in the first quarter of 2020 [9], great efforts including mask wearing, social distancing, vaccinations, test and trace strategies continue to struggle against COVID-19 [10–12]. Not only have these mitigation measures effectively maintained containment of COVID-19, but also they have impacted the prevalence of hospitalizations for neonatal infectious diseases.

The aims of the present study were to analyze the trend of hospitalizations for neonatal infectious diseases at a tertiary academic hospital and to assess impact of COVID-19 pandemic and associated prevention and control measures on infectious disease hospitalizations of neonates.

## Methods

### Study design and patients’ data

A hospital based cross-sectional study was conducted by collecting epidemiological data on hospital admissions for infectious diseases in neonatal inpatients at a tertiary academic hospital (the Children’s Hospital, Zhejiang University School of Medicine) from January 2015 to December 2020. The age of patients who required hospitalization with principal diagnoses of infectious diseases in the data set ranged from 0 to 28 days. Our institution is a national clinical research center for child health and a tertiary hospital of pediatric healthcare that has approximately 3.5 million outpatient visits and 81,000 inpatient admissions every year. The prevalence of infectious disease hospitalizations of neonates has been impacted by COVID-19 pandemic and corresponding containment measures implemented since January 2020.

### Protocol

The study protocol was approved by the ethics committee of the Children’s Hospital, Zhejiang University School of Medicine (IRB/EC Ref No. 2021-IRB-166). All of the clinical data included in this study were fully anonymized and could no longer be retraced. The need for individual informed consent was waived by the ethics committee because of the retrospective nature and anonymity of the study.

#### Inclusion criteria

Included were all neonatal inpatients with: (i) admission age ranged from 0 to 28 days AND (ii) principal diagnoses of infectious diseases AND (iii) who were admitted from January 2015 to December 2020.

#### Exclusion criteria

Excluded were neonatal inpatients with: (i) repetitive records OR (ii) lack of medical information (eg, length of hospital stay ≤ 2 days) OR (iii) non-infectious cases of newborn.

The variables collected for analysis included date of admission, date of discharge, length of stay, age, gender and principal diagnosis. An early newborn is defined as a newborn with postnatal age of less than 7 days and a late newborn is defined as a neonate with postnatal age of beyond 7 days. The diagnoses were made according to the International Classification of Diseases, version 10 (ICD-10). To facilitate the effective analysis, the diagnoses were grouped by using the subcategories criteria into respiratory infection, digestive system infection, urinary tract infection, intracranial infection, systemic infection and other or not specified infection. The cases with infection of two or more subcategories were coded with the first principal diagnoses. Clinical data were collected to compare hospitalizations during four quarters of 2020 (COVID-19 cohort), corresponding with the impact of COVID-19 pandemic and associated containment measures, and the comparable 2015 to 2019 quarters (pre-COVID-19 cohort).

### Statistical analysis

The categorical variables were described as absolute and relative frequencies, and numerical variables were described as mean values and standard deviations (SD). Categorical variables were compared between the COVID-19 and pre-COVID-19 cohorts by means of Chi-square tests. *T*-test was applied to compare normally distributed continuous variables. The simple seasonal model and Winters’ additive model were set up to forecast the numbers of hospitalizations for neonatal infectious diseases and proportions of those to the total hospitalizations of neonates, respectively. All analyses were performed with SPSS Statistics for Windows (software version 26.0). All *P* values < 0.05 were considered statistically significant.

## Results

**Fig. 1:**
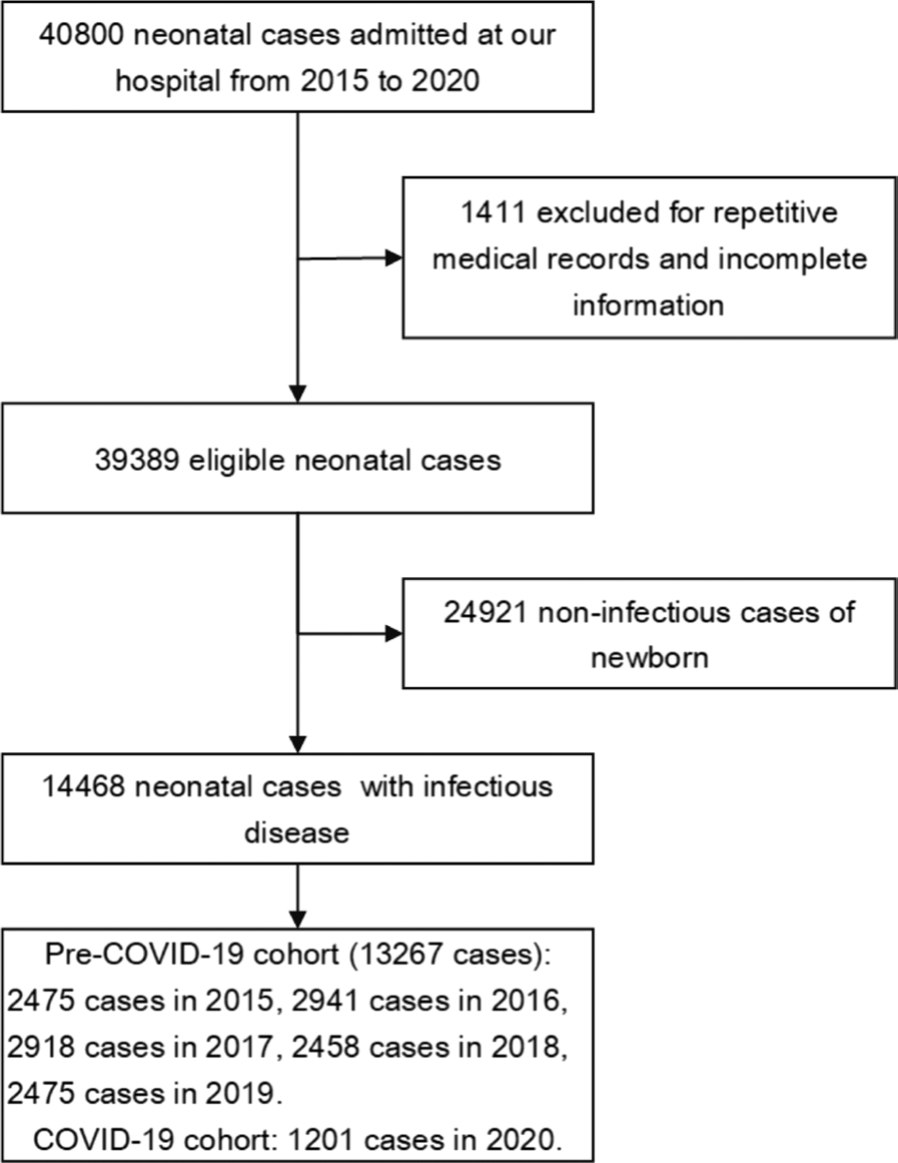
Study profile: A total of 40,800 cases of neonatal inpatients provided data to our study, and 14,468 of them were principally diagnosed with infectious diseases in the study, with 1201 cases in the COVID-19 cohort (2020) and 13,267 cases in the pre-COVID-19 cohort (from 2015 to 2019)

Figure 1 illustrates study participant selection and distribution in different years. Of the 40,800 cases of neonatal inpatients providing data to our study, 39,389 met inclusion criteria and contributed patient data to the study. 14,468 of them were principally diagnosed with infectious diseases (An Additional file 1 shows this in more detail), corresponding to a mean neonatal infectious disease hospitalization rate of 36.73%. There were 1201 cases in the COVID-19 cohort and 13,267 cases in the pre-COVID-19 cohort. All neonatal inpatients have received a nucleic acid test before admission in an effort to curb the spread of COVID-19 and ensure the safety of medical care in our hospital from January to December 2020, and all the results have been negative.

### Infectious disease hospitalizations of neonates by demographic group

**Fig. 2:**
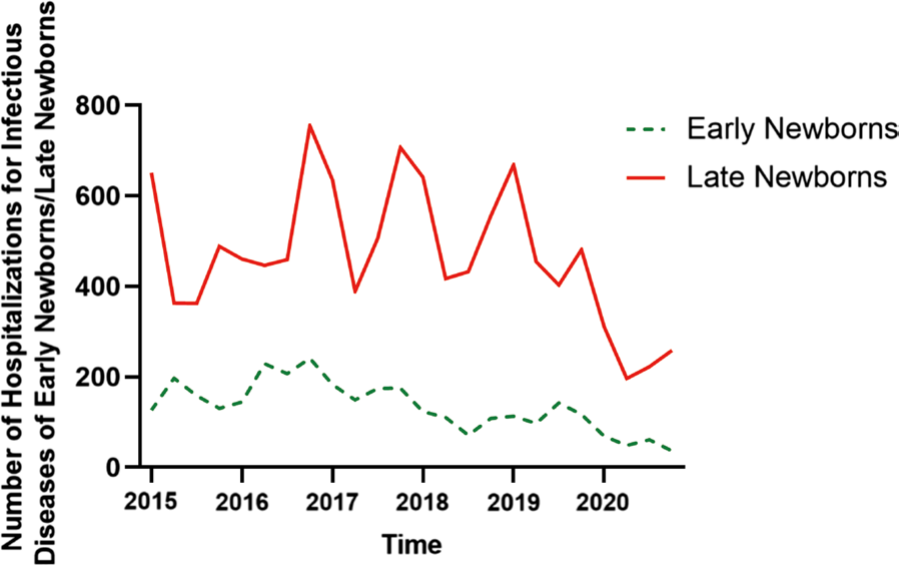
The numbers of hospitalizations for neonatal infectious diseases of early and late newborns. The numbers of both early and late newborns impacted by the COVID-19 pandemic significantly decreased in different quarters of 2020. Q: quarter

Categorization of ICD-10 codes for neonatal infectious disease cases by site of infection or sepsis is shown in Table 1. The demographic and clinical characteristics as well as the principal diagnoses are summarized in Table 2. This table also shows the comparisons of neonatal cases admitted for infectious diseases between the COVID-19 and pre-COVID-19 cohorts. The numbers of hospitalizations for neonatal infectious diseases of early newborns and late newborns in different quarters are shown in Fig. 2. In the hospitalizations for neonatal infectious diseases, late newborns are in the majority (64.8–87.8%). As shown in Fig. 2, the trend of the number of hospitalizations for infectious diseases of early newborns is basically similar to that of late newborns, and the numbers of quarterly hospitalizations for infectious diseases of both early newborns and late newborns were significantly lower in the COVID-19 cohort compared with the pre-COVID-19 cohort: early newborns (53.50 ± 14.53 vs 149.70 ± 8.31, *P* = 0.003), late newborns (246.75 ± 49.81 vs 513.65 ± 104.98, *P* = 0.003).

**Table 1:** Categorization of ICD-10 codes for infectious diseases by site of infection or sepsis used to evaluate infectious disease hospitalizations of neonates, the Children’s Hospital, Zhejiang University School of Medicine, 2015–2020

Category	ICD-10 code
Respiratory tract infection	A37.0, A37.9, A49.3, B08.5, B97.4, J00.x, J02.9, J04.0, J04.2, J06.9, J10.0, J10.1, J11.0, J11.1, J12.1, J12.2, J16.0, J16.8, J18.0, J18.9, J20.4, J20.9, J40.x, P22.2, P22.3, P23.0, P23.1, P23.6, P23.8, P23.9, P28.8, P35.0, P39.9, R05.x
Sepsis	A41.2, A41.9, A50.9, B00.9, B01.9, B08.4, B25.8, B25.9, B34.0, B34.9, P35.1, P36.3, P36.4, P36.9, P37.1, P58.2, R50.9, R65.2, R65.3
Gastric intestinal infection	A02.0, A04.9, A08.0, A08.4, A09.0, A09.9, K12.1, K35.0, K51.9, K52.9, P59.2, P77.x, R56.8
Dermatologic infection	B09.x, B35.0, H60.9, H61.9, K61.0, L00.X, L01.0, L02.2, L02.8, L02.9, L03.1, L03.3, L03.8, L03.9, L08.0, L08.9, L98.9, P15.8, P36.2, P38.x, P39.4, R22.9
Central nervous system infection	A39.0, A85.0, A86.x, A87.9, B00.3, B00.4, G00.8, G00.9, G03.9, G04.8, G06.0
Urinary tract infection	N34.2, N39.0, P39.3, R82.7
Other or not specified	A54.3, H00.0, H04.3, H10.9, H66.0, H66.4, H66.9, H70.9, I40.0, K11.2, K65.9, M00.9, N61.x, P39.0, P39.1, P39.8, P81.9, P83.4

**Table 2:** Infectious disease hospitalizations of neonates by demographic group, the Children’s Hospital, Zhejiang University School of Medicine, 2015–2020 (n = 14,468)

Total (14,468)	Pre-COVID-19 cohort (n = 13,267)	COVID-19 cohort (n = 1201)	P-value
Gender (male/female)	7468/5799	674/527	0.909
Postnatal age (days)	14.59 ± 8.44	15.30 ± 8.12	0.004
Age group (early newborn/late newborn)	3041/10226	214/987	< 0.001
Classification of diagnostic subcategories for infectious disease			
Respiratory infection	6251 (47.12%)	556 (46.29%)	0.587
Systemic infection	4390 (33.09%)	286 (23.81%)	0.000
Digestive system infection	1255 (9.46%)	145 (12.07%)	0.004
Skin infection	773 (5.83%)	96 (7.99%)	0.003
Intracranial infection	271 (2.04%)	44 (3.66%)	0.000
Urinary tract infection	106 (0.80%)	24 (2.00%)	0.000
Other or not specified infection	221 (1.67%)	50 (4.16%)	0.000
Length of hospital stay (days)	8.87 ± 7.60	8.65 ± 7.44	0.330

### Leading causes among infectious disease hospitalizations of neonates

**Fig. 3:**
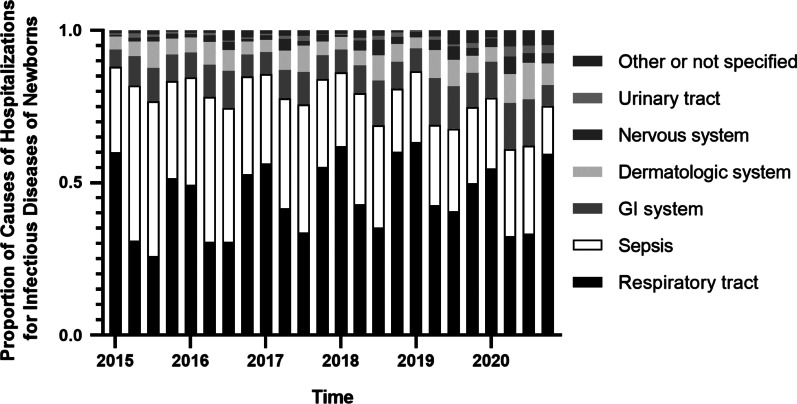
The proportions of causes of hospitalizations for neonatal infectious diseases. The leading causes of hospitalizations for neonatal infectious diseases included respiratory tract infections, sepsis, gastric intestinal infections and dermatologic infections in our study. Q: quarter

By analyzing the principal diagnoses of the data, it is found that there are many different causes of neonatal infectious diseases, with some of the most common ones being respiratory tract infections, sepsis, gastric intestinal infections, dermatologic infections, central nervous system infections and urinary tract infections (Fig. 3). With the proportions of causes changing in different quarters, the leading causes of hospitalizations for neonatal infectious diseases remain being respiratory tract infections (median ratio = 0.461, 95% CI 0.335–0.551), sepsis (median ratio = 0.292, 95% CI 0.263–0.361), gastric intestinal infections (median ratio = 0.095, 95% CI 0.078–0.118) and dermatologic infections (median ratio = 0.058, 95% CI 0.047–0.083) in our study.

### Infectious disease hospitalizations of neonates by the seasonality

**Fig. 4:**
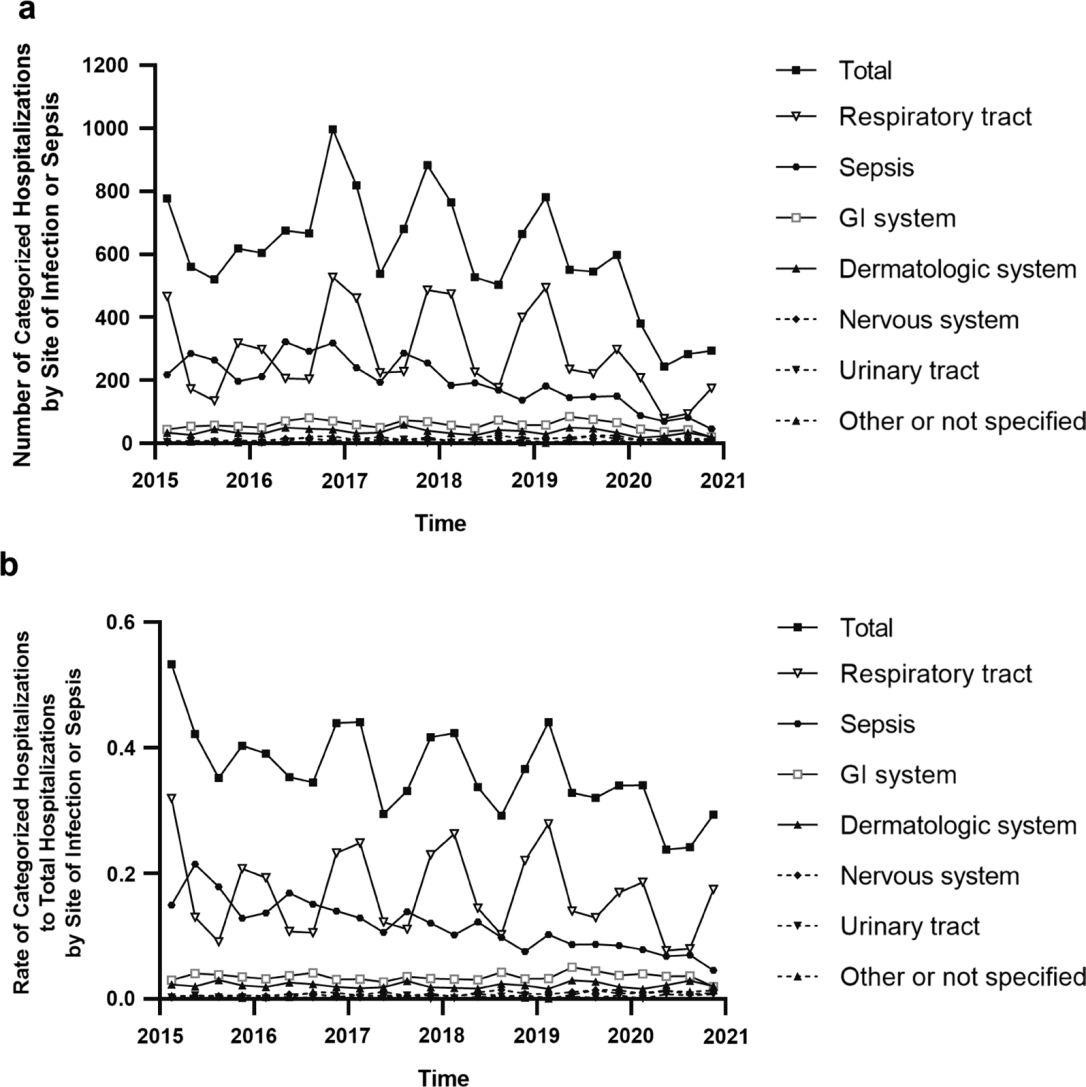
Infectious disease hospitalizations of neonates by the seasonality. **a** The number of categorized infectious disease hospitalizations of neonates. **b** The rate of categorized infectious disease hospitalizations of neonates

The observed numbers and the rates of hospitalizations for neonatal infectious diseases fluctuated in different quarters from January 2015 to December 2020 (Fig. 4). The total number or the overall rate of hospitalizations for neonatal infectious diseases in the first and fourth quarters was greater than that of hospitalizations for neonatal infectious diseases in the second and third quarters in each year (1362.67 ± 360.54 vs 1048.67 ± 279.23, *P* = 0.001; 8176/20020 vs 6292/19369, *P* < 0.001, respectively). Obviously, the seasonality of neonatal infectious disease hospitalizations could be observed. As it fluctuated in different quarters, the seasonality of respiratory tract infectious disease hospitalizations could also be obviously observed, with the total number or the overall rate in the first and fourth quarters greater than that in the other two quarters in our study (767.50 ± 197.78 vs 367.00 ± 109.00, *P* < 0.001; 4605/20020 vs 2202/19369, *P* < 0.001, respectively) (Fig. 4).

### Infectious disease hospitalizations of neonates by the impact of the COVID-19 pandemic

**Fig. 5:**
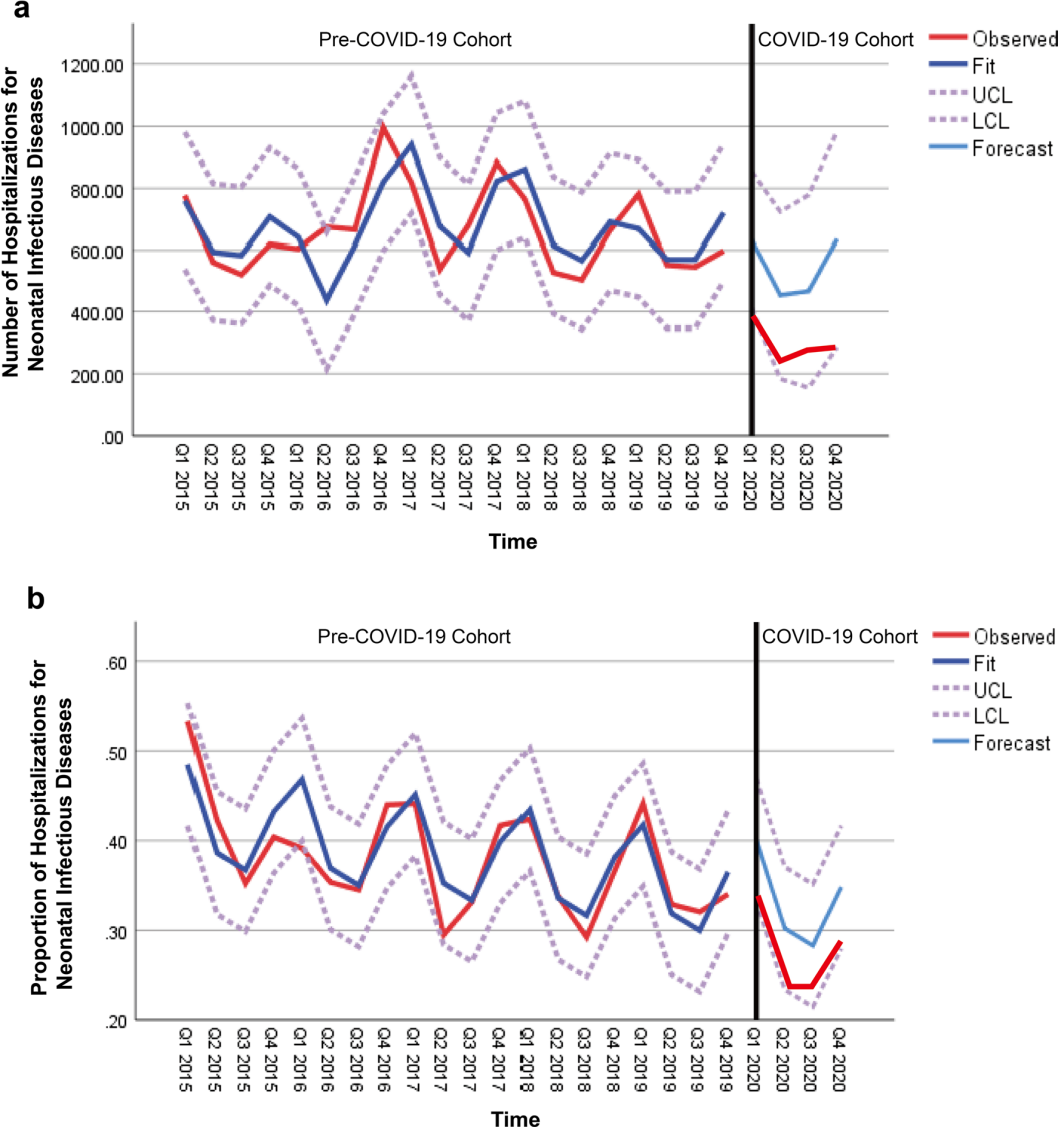
Infectious disease hospitalizations of neonates by the impact of the COVID-19 pandemic. Both the observed numbers (**a**) and proportions (**b**) of hospitalizations for neonatal infectious diseases in different quarters of the COVID-19 cohort significantly declined as compared with those forecasted. *UCL* upper confidence limit, *LCL* lower confidence limit, *Q* quarter

The simple seasonal model was applied to forecast the numbers of hospitalizations for neonatal infectious diseases in different quarters of 2020 with the data in the pre-COVID-19 cohort. As shown in Fig. 5a, the numbers of hospitalizations for neonatal infectious diseases in different quarters of the COVID-19 cohort significantly declined as compared with those forecasted (300.25 ± 57.33 vs 546.64 ± 100.43, *P* = 0.006).

The proportions of hospitalizations for neonatal infectious diseases to the total hospitalizations of neonates in different quarters of 2020 were forecasted by Winters’ additive model with the data in the pre-COVID-19 cohort. As shown in Fig. 5b, the proportions of hospitalizations for neonatal infectious diseases to the total hospitalizations of neonates in different quarters of the COVID-19 cohort significantly decreased as compared with those forecasted: the first quarter (0.34 vs 0.40, *P* = 0.002), the second quarter (0.24 vs 0.30, *P* = 0.001), the third quarter (0.24 vs 0.28, *P* = 0.024), and the fourth quarter (0.29 vs 0.35, *P* = 0.003).

## Discussion

This is a cross-sectional study on infectious disease hospitalizations of neonates during 2015–2020, focusing on the impact of COVID-19 pandemic on infectious disease hospitalizations. Although perinatological interventions and breakthroughs in neonatology have led to advances in reduction of neonatal morbidity and mortality [13, 14], infectious diseases remain to be the persistent threat of neonatal health, corresponding to a mean neonatal infectious disease hospitalization rate of over one third in our study. In the interest of public health, a lot of preventive measures have been introduced to curb the COVID-19 pandemic since January 2020. The neonates with infectious diseases have been dramatically impacted by these preventive measures. Thus, it is important to monitor infectious diseases of neonates to better understand their effect on the health care system and neonatal health.

Among neonatal factors that predispose neonates to infections, the status of the innate and adaptive immune response can play a significant role [15]. Early onset infectious diseases that compromise early newborns are mainly due to infections occurring during the antenatal or intrapartum period [16], which is usually described as “vertical transmission”. And late onset infectious diseases that compromise late newborns result from pathogens acquired from the environment after birth [17], which is also known as “horizontal transmission”. The significant decreases in the number of infectious disease hospitalizations in both the early and late newborn suggest the COVID-19 pandemic educational/governmental mitigation strategies can effectively reduce both vertical and horizontal transmissions of infectious disease in neonates, and the results could provide us with some beneficial enlightenment for prevention of community- and hospital-acquired infections of neonates. The COVID-19 pandemic educational/governmental mitigation strategies are certainly effective in the prevention of infectious diseases with horizontal transmissions, but the exact mechanism by which the COVID-19 mitigation strategies protect the neonates from vertically transmitted infections is unclear, which needs to be further investigated.

The respiratory tract infection was the most commonly listed cause of infection among infectious disease hospitalizations of neonates, and sepsis, gastric intestinal infections and dermatologic infections were listed as the other three main causes in infectious disease hospitalizations of neonates. These results are consistent with data documenting that the lower respiratory tract infection had the highest mean annual age-adjusted rate among infectious disease hospitalizations in the United States during 2001 to 2014 [18].

The prevalence of the infectious disease hospitalizations of neonates had significant seasonality with a high rate in winter and spring, especially respiratory tract infection hospitalizations of neonates. These results suggest considerable infectious disease hospitalizations of neonates that are likely to be due to climate factors that could alter the epidemiology of climate sensitive pathogens and host immune response. The association between climate factors and infectious disease is very complex and influenced by many factors such as socioeconomic status, viral prevalence, temperature, precipitation, relative humidity, and an increase in the level of air pollutants [19–21]. Further investigation into the relation between climate factors and neonatal infectious diseases is warranted.

We observed the numbers as well as the proportions of hospitalizations for neonatal infectious diseases in different quarters of 2020 dramatically declined. The main factor that drives such decreases in infectious disease hospitalizations of neonates is the impact of the COVID-19 pandemic. A previously unknown coronavirus was first discovered in samples from patients with pneumonia in December 2019, which triggered a global pandemic. The COVID-19 pandemic and its mitigation strategies have caused an unprecedented impact on all aspects of social and economic life, especially on human health and healthcare systems worldwide. Compliance of people with preventive measures and policies such as social distancing, face masking, sanitation, and isolation has played an important role in mitigating the pandemic as well as decreasing the prevalence of infectious disease of neonates. The mitigation strategies and preventive measures which are very effective in fighting the pandemic have contributed a lot in reducing the incidence of infectious disease of neonates. It is worth mentioning that public psychological factors should be considered. Although there have been no government recommendations or hospital policies to limit hospitalization admission or outpatient treatment during the COVID-19 pandemic, a few people might have avoided hospital visits and hospitalizations because they were worried about being infected by COVID-19 while going to hospital. Fortunately, there has been no significant increase in overall neonatal mortality in our province during the COVID19 pandemic according to the recent public data.

## Study limitations

This study had a few limitations. First, epidemiological data on hospital admissions for infectious diseases of neonates, the basis for this study, are subject to bias and other selective pressures that might affect principal diagnosis, such as reluctance to seek timely medical advice and treatment for fear of contracting the novel coronavirus in health care settings. Second, the study is a single-center based cross-sectional study with a relatively small sample. Third, we did not collect data on the pathogen type of the hospitalized newborn infants with infectious diseases. Finally, there were only inpatient data included in our study and no outpatient data.

## Conclusion

Despite the outbreak of the COVID-19 pandemic, the leading causes of hospitalizations for neonatal infectious diseases remain being respiratory tract infections, sepsis, gastric intestinal infections and dermatologic infections in our study. The numbers as well as the overall rates of hospitalizations for neonatal infectious diseases in different quarters of 2020 dramatically declined because of the impact of the COVID-19 pandemic and its mitigation measures.

## Supplementary Information

**Additional file 1.** Distribution of the number of infectious disease hospitalizations of neonates, the Children’s Hospital, Zhejiang University School of Medicine, 2015–2020.

## Data Availability

The used or analyzed data during the current study is available from the corresponding author on reasonable request.

## Abbreviations

COVID-19: Coronavirus disease 2019
ICD-10: International Classification of Diseases, version 10
